# 叙事护理对肺癌手术患者症状群管理及创伤后成长的应用研究

**DOI:** 10.3779/j.issn.1009-3419.2024.102.44

**Published:** 2025-01-20

**Authors:** Xinxing SUN, Yalin WANG, Wang LV, Linhai ZHU

**Affiliations:** ^1^310003 杭州，浙江大学医学院附属第一医院护理部; ^1^Nursing Department, The First Affiliated Hospital of Zhejiang University, Hangzhou 310003, China; ^2^310003 杭州，浙江大学医学院附属第一医院手术室; ^2^Operating Room, The First Affiliated Hospital of Zhejiang University, Hangzhou 310003, China; ^3^310003 杭州，浙江大学医学院附属第一医院普胸外科; ^3^Departmeng of General Thoracic Surgery, The First Affiliated Hospital of Zhejiang University, Hangzhou 310003, China

**Keywords:** 肺肿瘤, 叙事护理, 焦虑, 抑郁, 症状群, 创伤后成长, Lung neoplasms, Narrative care, Anxiety, Depression, Symptom clusters, Post-traumatic growth

## Abstract

**背景与目的:**

叙事护理作为实现高质量护理的一门新兴学科逐渐兴起，临床中可见于改善精神分裂症、抑郁症及慢性病管理等研究，但在肺癌手术患者中的应用鲜少报道。本研究旨在研究叙事护理模式对提升手术患者症状群管理及创伤后成长水平的效果，探讨其对促进肺癌患者身心康复的临床优势。

**方法:**

便利抽样选取2024年7月至2024年10月就诊于浙江大学医学院附属第一医院行手术治疗的82例肺癌患者为研究对象，按照随机数字法分为对照组和观察组，各41例。对照组接受常规护理；观察组在此基础上于患者入院当天、术后3天和术后1周融入连续3次的叙事护理。干预前收集患者的一般资料，采用广泛性焦虑量表、匹兹堡睡眠质量指数、中文版肺癌患者生活质量评估量表及中文版创伤后成长评定量表对其进行测评，并在每次叙事干预后对两组患者的情况再次进行测评，比较两组患者三阶段的得分情况。

**结果:**

术后3天、术后1周患者焦虑、睡眠、生活质量及创伤后成长水平得分，观察组均优于对照组，差异均具有统计学意义（*P*<0.05）。组内结果显示，观察组与对照组在术前、术后3天、术后1周焦虑评分均逐渐下降；术前、术后3天、术后1周创伤后成长评分均逐渐增加；但由于手术的应激及术后不适等原因，两组患者术后3天睡眠评分均高于术前和术后1周，术后1周睡眠评分均明显低于术前；术后3天的生存质量评分均低于术前和术后1周，术后1周生存质量评分均高于术前，差异均具有统计学意义（*P*<0.05）。

**结论:**

将叙事护理应用于肺癌手术患者中，有利于减轻围手术期症状群的困扰，帮助患者实现创伤后成长，提升其心理社会适应能力及生存质量。

## Abstract (en)

**Background and objective:**

Narrative care is emerging as a new discipline to achieve high-quality nursing. It can be seen in clinical studies on improving the management of schizophrenia, depression and chronic diseases, but its application in surgical patients with lung cancer is rarely reported. The aim of this study was to study the effect on improving symptom cluster management and post-traumatic growth of surgical patients through narrative care model, and to explore its clinical advantages in promoting physical and mental rehabilitation of lung cancer patients.

**Methods:**

A total of 82 patients with lung cancer who underwent surgical treatment in the First Affiliated Hospital of Zhejiang University from July 2024 to October 2024 were selected as the study objects by convenience sampling, and randomly divided into the control group and observation group according to random number method, with 41 cases in each group. The control group received routine nursing; On this basis, the observation group was integrated into three consecutive narrative nursing sessions on the day of admission, 3 days after surgery and 1 week after surgery. After collecting the general data of the patients before intervention, the Generalized Anxiety Scale, the Pittsburgh Sleep Quality Index, the Chinese Version of the Lung Cancer Patients Quality of Life Assessment Scale and the Chinese Version of the Post-traumatic Growth Assessment Scale were used. After each narrative intervention, the two groups of patients were assessed again, and the scores of the two groups were compared at the three stages.

**Results:**

The scores of anxiety, sleep, quality of life and post-traumatic growth level of the patients on the 3 days and 1 week postoperatively were better in the observation group than in the control group, and the differences were all statistically significant (*P*<0.05). In addition, the results within the groups showed that the anxiety scores of the observation group and the control group gradually decreased in the preoperative period, 3 days and 1 week postoperatively; the post-traumatic growth scores gradually increased in the preoperative period, 3 days and 1 week postoperatively; however, due to the stress of the surgery and postoperative discomfort, the sleep scores of the patients of the two groups on 3 days postoperatively were higher than those in the preoperative period and 1 week postoperatively, and the sleep scores of the patients of 1 week postoperatively were significantly lower than those in the preoperative period; and the quality of life of the patients on the 3 days postoperatively scores were lower than preoperative period and 1 week postoperatively, and the quality of survival scores in 1 week postoperatively were higher than the preoperative period, and the differences were statistically significant (*P*<0.05).

**Conclusion:**

The application of narrative nursing to lung cancer surgery patients is conducive to alleviating perioperative symptom clusters, helping patients achieve post-traumatic growth, and enhancing their psychosocial adaptability and quality of survival.

根据全球流行病学数据^[1]^显示，肺癌的发病率处于所有癌症的首位，且中国肺癌的发病率和死亡率呈现日益上升的趋势。目前外科手术切除是治疗肺癌的重要手段^[2]^，然而，肺癌手术患者不仅承受疾病本身的困扰，还容易出现焦虑、抑郁等情绪，伴随失眠、疼痛及疲乏^[3]^，这可能与癌症确诊对患者产生的重大冲击及手术等相关治疗对患者造成的创伤有关，这些症状之间存在相互协同的作用，以症状群的形式存在^[4]^，我们称为情绪-睡眠障碍症状群及疼痛-疲乏症状群，未予缓解的症状群又进一步刺激机体，导致手术相关并发症发生率的上升，严重影响患者术后生存质量及对预后的信心^[5,6]^。一项针对肺癌手术患者症状群的研究^[7]^显示，术后2周内患者疼痛、睡眠不安、疲乏、情绪不佳的发生率分别为75.3%、59.4%、54.0%和47.5%，因此，提升临床护理中症状群管理效果对于患者身心状态的改善意义重大。创伤后成长指个体在经历创伤性事件或逆境后产生的与成长相关的一系列积极心理变化^[8]^。有文献^[9]^显示，提高癌症患者创伤后成长水平可以提升其幸福感及治疗依从性。然而，目前国内外针对手术治疗的肺癌患者围手术期症状群的管理模式仍然缺乏。临床亟需构建基于循证的、系统化的、针对性强的护理管理策略，以促进患者术后康复，帮助其实现创伤后成长。

叙事护理疗法是指把积极心理学中叙事治疗的理念运用到临床护理中的一种心理护理模式^[10]^。国外相关研究^[11]^表明，具备叙事能力的护士通过故事倾听、吸收患者内在担忧的问题及痛苦经历来帮助患者抒发自身的情感，有助于患者减轻内心痛苦，培养正向情绪，从而产生源源不断向上的动力，最终利于疾病预后。目前国内叙事护理的应用可见于改善严重精神障碍或抑郁症患者的心理状态、慢性病管理等^[12]^，但在外科手术患者中的应用较少。在如今外科快速康复模式广泛应用的同时，如何在较短住院期间保证优质护理质量仍值得关注。本研究拟探究叙事护理模式对肺癌手术患者症状群管理及创伤后成长的影响，以期改善患者围手术期的预后，提高其生存质量。

## 1 资料与方法

### 1.1 一般资料

采用方便抽样法，选取2024年7月至2024年10月于浙江大学医学院附属第一医院行胸腔镜下肺癌根治术的82例患者为研究对象。依照前期预试验结果，本研究以焦虑为主要评价指标来计算样本量，样本量的计算公式为*n*=2[σ(Z_α/2_+Z_β_)]²÷(μ₁-μ₂)²中，设定α为0.05，β为0.10，查阅界值表后可得Z_α/2_=1.96，Z_β_=1.28；参考前期预试验情况，观察组焦虑干预前后的差值μ_1_=14.66，对照组焦虑评分干预前后的差值μ_2_=12.20，对照组焦虑的标准差σ=3.16。经过计算每组所需样本量为37例，考虑失访增加10%的病例，最终计算样本量为82例，按照随机数字法分为试验组和对照组，各41例。纳入标准：（1）年龄≥18岁；（2）首次确诊为原发性非小细胞肺癌（non-small cell lung cancer, NSCLC）并行肺癌切除术；（3）意识清楚、理解表达能力正常。排除标准：（1）其他癌症转移或复发肺癌；（2）术前接受过化疗；（3）发生非计划二次手术；（4）参加类似研究；（5）病情危重或存在严重功能障碍，无法配合完成沟通；（6）既往存在严重精神疾病史。剔除标准：整个围手术期中接受叙事护理中断≥1次者。本研究已通过浙江大学医学院附属第一医院医学伦理委员会审查（审查号：IIT20240603B）。所有研究对象均知晓相关情况并同意且自愿参加研究。

### 1.2 方法

#### 1.2.1 干预方法

对照组患者接受常规心理护理，主要涵盖：在入院当天，责任护士主动介绍，对患者进行肺癌手术相关知识宣教，在患者住院期间耐心倾听患者的诉求，指导患者掌握一些常规放松心情的方式，帮助患者树立康复的信念。对主要照顾者进行围手术期护理知识教育并予以安抚，同时指导照顾者密切观察患者的心理反应并及时与观察小组人员沟通。观察组在此基础上于患者入院当天、术后3天及术后1周实施引导叙事、分离解构和重塑自我3个阶段的叙事护理干预。

#### 1.2.2 成立叙事护理小组

选取7名医护人员成立叙事护理小组，成员包括1名精神卫生科主任医师，负责叙事干预过程中的沟通技巧指导；1名拥有国家二级心理咨询师证书的副主任护师，负责叙事方案的拟定及全程质量把控；1名具备丰富临床护理经验的护士长，负责对入组患者进行筛查以及建立关系；3名具有8年以上专科经验、中级以上职称的护士（均接受过叙事护理相关知识培训）统一指导用语、资料收集及叙事护理实践，1名手术室专科护士负责数据整理及分析。

#### 1.2.3 叙事方案制定及实践

本次叙事干预方案在叙事医学疗法理论模型^[13,14]^基础上，结合文献回顾及专家咨询而形成，并邀请了3名心理护理领域的专家对方案进行了指导和修正。在叙事实践开展前，选取10例患者进行预试验，根据预试验效果修订叙事护理的实践内容，形成最终叙事实施方案。每次叙事护理干预为35-45 min。

首次叙事于手术患者入院当天面对面进行，以“患者具体问题的解释和发展”为基础引导叙事：在患者一般资料基础上结合其性格特征展开交流，对性格内向的患者多采用诱导式的问题，主动深入其内心，鼓励其分享叙事。对于性格开朗外向的患者，赋予其主导权，把握交谈的主题，做一个耐心的聆听者并予以回应与鼓励。为每位患者建立叙事病历，记录患者叙事内容，通过开展肺癌手术等知识的宣教进行主动沟通，逐步与患者建立信任关系。不直接询问患者目前的困扰，有过渡地鼓励患者表达自己内心的真实想法，并循序渐进地引导患者大胆地发泄情绪并勇于诉说自己的烦恼，不评论对错，而是予以尊重和回应。当患者情绪低落时，给予积极的正面反馈，让患者觉得他的烦恼和痛苦是可以被人理解的，并不是孤立无援。

第二次叙事于术后3天患者体力及身体机能适当恢复时进行，围绕“每个人都是自身生命故事的专家”为主题分离自身矛盾：帮助患者理解问题是存在于个体之外的，人本身与问题并不等同。借助患者的讲述开启对话的空间，了解肺癌是如何发生的以及患者对手术治疗的态度，帮助患者“外化”影响自身的严重问题，将患者与问题分开，使问题具体化，为患者提供第三视角来看待问题及其影响。针对术后疼痛困扰严重的患者，指导其通过听音乐、看视频等方式转移注意力，或引导患者回顾和挖掘以往生活经历中的困难以及自己是如何克服解决的。同时放大逆境事件背后的积极意义，帮助患者接纳疾病，找到自己的闪光点，认可自己的各项潜力，从而激发患者的内在动力。

第三次叙事于术后1周患者出院后，小组成员采用远程网络平台视频或电话的方式进行，以“为生命赋能，达成生命的流动与蓬勃”为目标反思自我：叙事成员指导患者回想叙事过程，交流自己的收获及感悟。关心及询问患者肺部康复情况，鼓励患者与朋友和爱人分享疾病经历，帮助患者提升战胜疾病的信念，寻找未被自我叙事纳入的有力量的经验。关注生活中的美好事件，扩大患者的社会支持来源，强化患者的信心，培养积极向上的态度。

#### 1.2.4 叙事护理质量控制

本次叙事干预方案由具备心理咨询师资质的研究者完成，确保干预的科学性。研究期间，每个阶段相互递进、层层渗透，每次叙事完成后由叙事小组成员记录经过并思考干预时患者的状态及反应、了解到的内容及遇到的问题。每周固定时间组织小组会议，小组成员针对叙事过程进行讨论，交流叙事过程中发现的问题，由精神卫生科医生及资深心理咨询师对下一步干预进行指导，通过反思不断提升小组成员的叙事实践能力。

### 1.3 研究工具

#### 1.3.1 一般资料调查表

调查表由叙事小组成员设计，包括性别、年龄、家庭、社会背景、文化层次、住址、肺癌分期等临床资料。

#### 1.3.2 广泛性焦虑量表（Generalized Anxiety Disorder, GAD-7）^[15]^

由Spitzer等编制，国内外研究均显示GAD-7对焦虑情绪筛查具有较高的信效度，该量表共7个条目，采用0-3级评分，总分范围0-21分，Cronbach's α系数为0.859。0-4分为无焦虑，轻度、中度和重度焦虑的得分分别为5-9、10-14和15分及以上。

#### 1.3.3 匹兹堡睡眠质量指数（Pitsburgh Sleep Quality Index, PSQI）^[16]^

该量表包含7个维度，包括入睡潜伏时间、睡眠效率、主观睡眠质量、睡眠时间、睡眠干扰因素、安眠药物使用情况及白天功能障碍，用于评定患者的睡眠情况，每个维度按0-3等级评分，总分范围0-21分，PSQI总分≤7分为睡眠质量基本满意，PSQI总分>7分为睡眠质量差。

#### 1.3.4 肺癌患者生存质量测定量表（Functional Assessment of Cancer Therapy-Lung, FACT-L）中文版

该量表由万崇华等^[17]^汉化，包含社会/家庭状况、情感状况、功能状况、生理状况及附加关注状况6个领域共38个条目，各条目采用Likert 5级评分法。生活质量总分为各维度得分之和，总分及各维度得分越高说明肺癌患者生活质量越好。该量表的信效度经过了严格的检验，总量表的Cronbach's α系数为0.841。

#### 1.3.5 简体中文版创伤后成长评定量表（Chinese-Post traumatic Growth Inventory, C-PTGI）

此量表由汪际等^[18]^编制，涵盖5个领域共20个条目，这5个领域分别是：个人力量、新的可能性、与他人关系、自我转变、人生感悟。总分为0至100分，分数越高意味着创伤后成长水平越高，总量表以及各领域的Cronbach's α系数处于0.611至0.874之间。

#### 1.3.6 资料收集方法

开展研究前资深精神卫生科主任医师对叙事小组成员采用统一指导语培训。小组成员根据纳入排除标准筛选研究对象后，于患者入院当天面对面向患者详细陈述研究的目的、意义及注意事项，征得其知情同意后方可实施。首先采用一般资料调查表收集患者的基本资料，在干预前评价两组患者焦虑、失眠、生活质量及创伤后成长情况，并于术后3天及术后1周的叙事干预后再次评价两组患者的焦虑、失眠、生活质量及创伤后成长水平，比较两组患者三阶段的得分。对不识字或不方便填写调查表的患者，由叙事成员询问并代为填写。在数据分析之前，所有数据由双人录入，以保证数据的准确性。

### 1.4 统计学方法

采用SPSS 26.0软件予以分析，计量资料采用均数±标准差（Mean±SD）表示，组间对比采用独立样本*t*检验，组内叙事干预前后比较采用单因素重复测量方差分析，两两比较采用*LSD*法。分类计数资料使用例数（%）表示，组间比较采用*χ*^2^检验。*P*<0.05为差异具有统计学意义。

## 2 结果

### 2.1 一般资料

干预期间，观察组1例主动退出研究，对照组失访1例。最终观察组40例和对照组40例完成研究，年龄24-82（54.00±15.13）岁。观察组中，年龄27-78（54.33±13.81）岁，男性15例、女性25例；对照组中，年龄24-82（53.68±16.50）岁，男性19例、女性21例。两组患者的临床一般资料见表1。两组患者的性别、年龄、家庭、社会背景、文化层次、住址、肺癌分期等经*χ*^2^检验结果显示，差异均无统计学意义（*P*>0.05）。

**表1:** 两组患者的临床特征

General information	Observation group (n=40)	Control group (n=40)	*χ*^2^	P
Gender			0.818	0.366
Male	15 (37.5%)	19 (47.5%)		
Female	25 (62.5%)	21 (52.5%)		
Age			0.205	0.651
<50 yr	16 (40.0%)	18 (45.0%)		
≥50 yr	24 (60.0%)	22 (55.0%)		
Education level			1.072	0.784
Primary and below	13 (32.5%)	11 (27.5%)		
Junior high school	8 (20.0%)	9 (22.5%)		
High school or secondary school	5 (12.5%)	8 (20.0%)		
College and above	14 (35.0%)	12 (30.0%)		
Careers			0.355	0.837
Incumbency	20 (50.0%)	18 (45.0%)		
Retirement	7 (17.5%)	9 (22.5%)		
Out of work	13 (32.5%)	13 (32.5%)		
Marital status			0.182	0.913
Married	29 (72.5%)	29 (72.5%)		
Unmarried	5 (12.5%)	6 (15.0%)		
Divorced/widowed	6 (15.0%)	5 (12.5%)		
Address			0.220	0.896
Municipalities	12 (30.0%)	13 (32.5%)		
Townships	15 (37.5%)	13 (32.5%)		
Countryside	13 (32.5%)	14 (35.0%)		
Pathological stage			0.220	0.639
Stage I	13 (32.5%)	15 (37.5%)		
Stage II and above	27 (67.5%)	25 (62.5%)		

### 2.2 观察指标

观察组与对照组三阶段各评分比较见表2。观察组和对照组患者三阶段评分经独立样本*t*检验结果显示，两组患者术前焦虑、睡眠指数、生存质量和创伤后成长评分差异均无统计学意义（*P*>0.05）。观察组术后3天、术后1周焦虑、睡眠、生存质量和创伤后成长评分均明显优于对照组，差异均有统计学意义（*P*<0.05）。组内手术前后的评分经单因素重复测量方差分析所得结果表明，观察组与对照组术后3天、术后1周焦虑评分均低于术前，术后1周焦虑评分均明显低于术后3天；术后3天睡眠指数评分均高于术前和术后1周，术后1周睡眠评分均明显低于术前；术后3天生存质量评分均低于术前和术后1周，术后1周生存质量评分明显高于术前；术后3天和术后1周患者的创伤后成长评分均高于术前，术后1周创伤后成长评分均高于术后3天，差异均有统计学意义（*P*<0.05）。

**表2:** 两组三阶段各评分比较

Norm	Point of time	Observation group (n=40)	Control group (n=40)	t	P
GAD-7	Day of admission	11.03±4.40	11.30±4.22	0.285	0.776
	3 days after surgery	7.73±3.16^a^	9.78±4.17^a^	2.479	0.015
	1 week after surgery	5.35±2.35^ab^	8.03±3.08^ab^	4.372	<0.001
PSQI	Day of admission	10.05±2.68	10.07±2.95	0.040	0.968
	3 days after surgery	11.45±3.43^a^	14.20±3.50^a^	3.553	0.001
	1 week after surgery	6.83±2.10^ab^	9.05±2.44^ab^	4.374	<0.001
FACT-L	Day of admission	100.28±14.52	99.13±15.10	0.347	0.729
	3 days after surgery	92.50±12.79^a^	83.90±11.85^a^	3.120	0.003
	1 week after surgery	112.48±15.89^ab^	104.10±14.11^ab^	2.492	0.015
C-PTGI	Day of admission	65.18±9.15	65.55±9.90	0.176	0.861
	3 days after surgery	72.55±9.42^a^	68.10±9.63^a^	2.088	0.040
	1 week after surgery	80.47±9.02^ab^	74.18±8.61^ab^	3.195	0.002

^a^: Comparison with the point of “day of admission”; ^b^: Comparison with the point of “3 days after surgery”.

GAD-7: Generalized Anxiety Disorder; PSQI: Pitsburgh Sleep Quality Index; FACT-L: Functional Assessment of Cancer Therapy-Lung; C-PTGI: Chinese-Post traumatic Growth Inventory.

## 3 讨论

### 3.1 叙事护理有利于提升肺癌手术患者症状群管理效果

不同于传统单向讲授的心理护理方式，叙事护理模式为患者提供述说机会，使患者充分表达现有的不适、困惑，聚焦当下，剖析解决问题，从而帮助患者建立正向情绪来应对各种应激事件^[19]^，包括肺癌围手术期所致的焦虑、失眠、疲乏感及疼痛等症状。受社会文化影响，大多数肺癌患者内心往往较为内敛、隐忍^[20]^，一方面因患病导致自卑、抑郁，担心他人无法共情自身的遭遇，因而不愿主动向他人袒露内心的真实情绪；另一方面，因疾病影响工作收入，手术及后续治疗费用等原因加重家庭经济困难，更在某种程度上增加了患者的心理负担^[21]^，使围手术期的症状群进一步加重。接受过叙事训练的护理人员不仅拥有专业的临床护理知识、临床判断能力，同时具备了良好的沟通能力和叙事护理决策力^[22]^，作为患者倾诉和宣泄自身负性感受的出口，可以更有效地帮助围手术期患者正视和引导自身的情绪。

### 3.2 叙事护理有利于提高肺癌手术患者创伤后成长水平

本研究根据叙事医学疗法理论模型制定，结合文献回顾及专家咨询，通过实践干预反复修正从而形成分阶段叙事主题访谈，该方案作为一种自我表露的方式，传递了患者潜在的内心世界，通过个人叙事增强了医护人员和照顾者对患者心理体验的了解，促进患者的自我认同，进而激发患者内心潜在的力量^[23]^，积极事件的回顾可增进患者对自我价值的肯定，增强患者创伤后自我成长水平^[24]^。由此可见，在科学技术和人工智能广泛应用的今天，有“温度”的护理仍无法被其他形式取代，因其能为患者提供更为个性化、充满人文关怀的护理照顾。

### 3.3 肺癌手术患者的身心变化是一个动态的过程

我们根据两组患者组内手术前后评分分析结果可知，两组患者睡眠质量指数3次评估值先升高后降低，肺癌患者生活质量评分的3次评估值呈现先降低后升高的趋势，分析其原因，该现象可能由于患者术后切口疼痛及围手术期住院治疗的不适应而导致，还表明肺癌手术患者自我调节能力及对症状的适应性随时间推移逐步提高。而在术后3天，观察组手术患者的睡眠及生活质量改善情况相较于对照组患者更为明显，证明叙事护理可以降低但不能完全消除肺癌患者的围手术期应激反应。因此，肺癌手术患者作为一类特殊人群，临床护理中更要注意手术这类应激性操作对患者身体和心理带来的影响和变化，不同患者因性格和身体耐受力的差别，对环境及身体变化的适应力和疼痛的耐受力各有不同^[25]^。在实际的叙事护理过程中，要有针对性地引导谈话的主题和内容，根据患者的术后身心变化及时调整叙事干预方案。

综上所述，叙事护理是护理学科人文属性的体现，也是护理作为一门独立的学科不断发展的产物。叙事护理理论同样需要叙事护理反复实践以丰富其内涵，然而，叙事护理在国内的临床实践尚处于起始阶段，推进其理论创新和临床管理是新生事物不断发展的必然选择。本研究仅收集了单中心数据，样本代表性受限，今后可考虑扩大样本量，开展多中心试验并延长追踪观察时间，深入探讨叙事护理模式对肺癌手术患者相关症状群的远期改善效果；未来研究也可纳入更多人口学及心理学指标，进一步探索提升肺癌患者创伤后成长的影响因素，并持续充实、细化叙事护理干预方案，增强干预效果。
